# Supplementary material for: AUXIN RESPONSE FACTOR 1 Acts as a Positive Regulator in the Response of Poplar to Trichoderma asperellum Inoculation in Overexpressing Plants
Source: Plants (Basel). 2020 Feb 19;9(2):272. doi: 10.3390/plants9020272 (PMC7076496; doi:10.3390/plants9020272)
Supplement: Supplementary file 1 [file plants-09-00272-s001.zip › supplementary material/Table S3 revised Round 2.docx]

**Table S3.** Data for Figure 5.

| **Gene name** | **Treatment** | **Relative expression of each gene in different compartments** | | |  |
| --- | --- | --- | --- | --- | --- |
|  |  | **ST** | **L** | **R** |  |
| *PdPapCOI1* | WT | 1.000±0.000b^1^ | 1.000±0.000b | 1.000±0.000cde |  |
|  | OX1 | 0.925±0.087bc | 0.636±0.111c | 1.352±0.110c |  |
|  | WT+Ta536 | 0.801±0.033cd | 0.862±0.092b | 1.202±0.124cd |  |
|  | OX1+Ta536 | 1.398±0.151a | 0.548±0.092cd | 0.779±0.108e |  |
|  | WT+Aa | 0.638±0.142de | 0.354±0.079e | 1.328±0.157c |  |
|  | OX1+Aa | 0.995±0.038b | 1.431±0.066a | 4.561±0.526a |  |
|  | WT+Ta536+Aa | 0.681±0.149de | 0.465±0.052cde | 0.843±0.085de |  |
|  | OX1+Ta536+Aa | 0.529±0.094e | 0.429±0.132de | 1.892±0.120b |  |
| *PdPapJAZ5* | WT | 1.000±0.000d | 1.000±0.000bc | 1.000±0.000d |  |
|  | OX1 | 2.869±0.278b | 1.053±0.157b | 2.086±0.135c |  |
|  | WT+Ta536 | 1.612±0.258cd | 0.744±0.097bc | 1.808±0.295c |  |
|  | OX1+Ta536 | 4.110±0.367a | 0.759±0.130bc | 1.194±0.068d |  |
|  | WT+Aa | 1.302±0.211cd | 0.326±0.050d | 1.771±0.160c |  |
|  | OX1+Aa | 2.729±0.331b | 2.511±0.238a | 9.494±0.455a |  |
|  | WT+Ta536+Aa | 1.866±0.420c | 0.649±0.150cd | 1.262±0.148d |  |
|  | OX1+Ta536+Aa | 0.778±0.200d | 0.800±0.133bc | 2.935±0.246b |  |
| *PdPapMYC2* | WT | 1.000±0.000c | 1.000±0.000b | 1.000±0.000c |  |
|  | OX1 | 0.725±0.121cd | 0.956±0.046bc | 1.389±0.085b |  |
|  | WT+Ta536 | 1.626±0.229b | 0.949±0.090bc | 1.373±0.199b |  |
|  | OX1+Ta536 | 2.291±0.182a | 0.933±0.043bc | 0.519±0.014d |  |
|  | WT+Aa | 0.761±0.113cd | 0.777±0.135c | 0.538±0.019d |  |
|  | OX1+Aa | 1.392±0.176b | 2.849±0.241a | 5.065±0.264a |  |
|  | WT+Ta536+Aa | 0.879±0.072cd | 0.881±0.065bc | 0.438±0.082d |  |
|  | OX1+Ta536+Aa | 0.557±0.136d | 0.978±0.038bc | 0.954±0.065c |  |
| *PdPapNPR1* | WT | 1.000±0.000e | 1.000±0.000e | 1.000±0.000e |  |
|  | OX1 | 1.321±0.042c | 1.439±0.186d | 1.606±0.108de |  |
|  | WT+Ta536 | 1.618±0.113b | 2.083±0.109b | 3.830±0.657b |  |
|  | OX1+Ta536 | 1.968±0.103a | 1.714±0.113c | 1.273±0.081e |  |
|  | WT+Aa | 1.109±0.056de | 1.156±0.036e | 1.614±0.028de |  |
|  | OX1+Aa | 1.281±0.152cd | 3.163±0.163a | 7.691±0.571a |  |
|  | WT+Ta536+Aa | 2.007±0.056a | 2.085±0.113b | 1.970±0.217cd |  |
|  | OX1+Ta536+Aa | 0.551±0.052f | 1.505±0.092d | 2.284±0.251c |  |
| *PdPapTGA* | WT | 1.000±0.000a | 1.000±0.000b | 1.000±0.000b |  |
|  | OX1 | 0.913±0.056a | 0.643±0.049c | 0.697±0.119c |  |
|  | WT+Ta536 | 0.612±0.087b | 0.443±0.116d | 1.066±0.067b |  |
|  | OX1+Ta536 | 1.000±0.047a | 0.459±0.069d | 0.414±0.016d |  |
|  | WT+Aa | 0.088±0.013d | 0.182±0.001f | 0.347±0.032d |  |
|  | OX1+Aa | 0.701±0.097b | 1.141±0.040a | 2.936±0.152a |  |
|  | WT+Ta536+Aa | 0.113±0.025d | 0.307±0.077e | 0.280±0.006d |  |
|  | OX1+Ta536+Aa | 0.295±0.045c | 0.520±0.105d | 0.596±0.076c |  |
| *PdPapPR1* | WT | 1.000±0.000b | 1.000±0.000ab | 1.000±0.000c |  |
|  | OX1 | 0.334±0.075c | 0.545±0.105c | 3.140±0.412bc |  |
|  | WT+Ta536 | 0.883±0.183b | 0.820±0.093b | 4.527±1.049b |  |
|  | OX1+Ta536 | 0.388±0.071c | 0.238±0.025d | 1.421±0.224c |  |
|  | WT+Aa | 1.116±0.218b | 0.404±0.029cd | 3.299±0.189bc |  |
|  | OX1+Aa | 0.383±0.072c | 1.134±0.095a | 25.040±3.579a |  |
|  | WT+Ta536+Aa | 1.658±0.168a | 0.445±0.065cd | 0.866±0.146c |  |
|  | OX1+Ta536+Aa | 0.394±0.118c | 0.979±0.089ab | 4.289±0.364b |  |
| *PdPapARF1* | WT | 1.000±0.000c | 1.000±0.000e | 1.000±0.000c |  |
|  | OX1 | 1.391±0.015b | 2.101±0.088b | 1.525±0.131bc |  |
|  | WT+Ta536 | 0.843±0.070d | 1.315±0.073d | 2.115±0.384bc |  |
|  | OX1+Ta536 | 3.192±0.076a | 1.813±0.208c | 1.145±0.057c |  |
|  | WT+Aa | 0.408±0.040e | 0.702±0.162f | 1.607±0.262bc |  |
|  | OX1+Aa | 1.306±0.051b | 3.648±0.126a | 8.444±0.678a |  |
|  | WT+Ta536+Aa | 0.489±0.113e | 0.732±0.127f | 1.462±0.161c |  |
|  | OX1+Ta536+Aa | 0.852±0.066d | 1.324±0.028d | 2.629±0.390b |  |
| *PdPapARF6* | WT | 1.000±0.000e | 1.000±0.000b | 1.000±0.000b |  |
|  | OX1 | 6.798±1.168a | 0.172±0.025d | 0.209±0.028d |  |
|  | WT+Ta536 | 1.556±0.192de | 0.551±0.130bcd | 0.600±0.167bcd |  |
|  | OX1+Ta536 | 2.633±0.722bcd | 0.726±0.152bc | 0.411±0.108cd |  |
|  | WT+Aa | 2.754±0.401bc | 0.842±0.220bc | 0.998±0.061b |  |
|  | OX1+Aa | 3.270±0.636b | 2.607±0.335a | 2.724±0.381a |  |
|  | WT+Ta536+Aa | 2.307±0.528bcd | 0.595±0.092bcd | 0.467±0.026cd |  |
|  | OX1+Ta536+Aa | 2.074±0.297cde | 0.349±0.034cd | 0.799±0.183bc |  |
| *PdPapARF8* | WT | 1.000±0.000c | 1.000±0.000d | 1.000±0.000d |  |
|  | OX1 | 2.204±0.269b | 2.387±0.370c | 4.075±0.082bc |  |
|  | WT+Ta536 | 0.701±0.088c | 1.156±0.071d | 2.100±0.429cd |  |
|  | OX1+Ta536 | 2.088±0.121b | 3.677±0.870b | 2.721±0.183cd |  |
|  | WT+Aa | 0.834±0.205c | 0.766±0.164d | 1.719±0.077cd |  |
|  | OX1+Aa | 3.364±0.618a | 7.633±1.153a | 22.599±3.509a |  |
|  | WT+Ta536+Aa | 0.505±0.059c | 0.466±0.101d | 1.057±0.142d |  |
|  | OX1+Ta536+Aa | 2.109±0.362b | 3.061±1.105bc | 6.128±1.223b |  |
| *PdPapGH3.5* | WT | 1.000±0.000c | 1.000±0.000c | 1.000±0.000b |  |
|  | OX1 | 1.247±0.127b | 0.652±0.074d | 0.868±0.100b |  |
|  | WT+Ta536 | 1.387±0.074a | 1.279±0.118b | 0.947±0.042b |  |
|  | OX1+Ta536 | 0.516±0.068e | 0.675±0.004d | 0.521±0.123cd |  |
|  | WT+Aa | 0.438±0.051ef | 1.496±0.091a | 0.641±0.045c |  |
|  | OX1+Aa | 0.856±0.025d | 1.083±0.088c | 1.942±0.229a |  |
|  | WT+Ta536+Aa | 0.849±0.102d | 0.587±0.094d | 0.255±0.055e |  |
|  | OX1+Ta536+Aa | 0.354±0.074f | 0.596±0.032d | 0.411±0.053de |  |
| *PdPapGH3.6* | WT | 1.000±0.000d | 1.000±0.000d | 1.000±0.000e |  |
|  | OX1 | 5.983±1.173c | 1.986±0.283c | 5.553±1.092bc |  |
|  | WT+Ta536 | 1.070±0.126d | 0.406±0.065e | 1.739±0.337de |  |
|  | OX1+Ta536 | 11.165±1.588a | 2.473±0.381b | 4.150±0.462cd |  |
|  | WT+Aa | 0.624±0.105d | 0.235±0.014e | 2.260±0.460de |  |
|  | OX1+Aa | 8.979±0.630b | 5.311±0.569a | 21.081±3.881a |  |
|  | WT+Ta536+Aa | 1.434±0.414d | 0.329±0.052e | 0.670±0.079e |  |
|  | OX1+Ta536+Aa | 5.520±0.922c | 1.044±0.221d | 7.242±0.837b |  |
| *PdPapPIN1-1* | WT | 1.000±0.000e | 1.000±0.000b | 1.000±0.000ef |  |
|  | OX1 | 3.243±0.625c | 0.436±0.050d | 1.316±0.067d |  |
|  | WT+Ta536 | 0.862±0.149e | 1.598±0.111a | 2.126±0.273b |  |
|  | OX1+Ta536 | 4.785±0.378b | 0.957±0.045b | 1.214±0.197de |  |
|  | WT+Aa | 1.187±0.147e | 0.837±0.053b | 0.917±0.008f |  |
|  | OX1+Aa | 6.166±0.475a | 1.457±0.168a | 3.628±0.052a |  |
|  | WT+Ta536+Aa | 2.537±0.258d | 0.411±0.050d | 1.117±0.058def |  |
|  | OX1+Ta536+Aa | 3.043±0.520cd | 0.661±0.136c | 1.890±0.111c |  |
| *PdPapPIN1-2* | WT | 1.000±0.000e | 1.000±0.000cd | 1.000±0.000d |  |
|  | OX1 | 1.026±0.020e | 0.468±0.046e | 1.116±0.066d |  |
|  | WT+Ta536 | 0.527±0.126f | 1.800±0.059b | 1.806±0.295b |  |
|  | OX1+Ta536 | 3.428±0.395b | 1.878±0.085b | 1.247±0.123cd |  |
|  | WT+Aa | 1.104±0.080e | 1.144±0.083c | 1.302±0.204cd |  |
|  | OX1+Aa | 4.825±0.391a | 2.471±0.334a | 2.970±0.303a |  |
|  | WT+Ta536+Aa | 2.501±0.120d | 0.963±0.076cd | 0.459±0.030e |  |
|  | OX1+Ta536+Aa | 2.921±0.021c | 0.875±0.112d | 1.659±0.347bc |  |
| *PdPapLAX3* | WT | 1.000±0.000e | 1.000±0.000d | 1.000±0.000cd |  |
|  | OX1 | 3.887±0.772b | 1.777±0.140b | 1.502±0.233c |  |
|  | WT+Ta536 | 0.997±0.104e | 0.990±0.041d | 0.765±0.104d |  |
|  | OX1+Ta536 | 7.424±0.413a | 1.535±0.024c | 1.177±0.065cd |  |
|  | WT+Aa | 0.900±0.068e | 0.344±0.041f | 0.850±0.176cd |  |
|  | OX1+Aa | 3.524±0.263b | 2.727±0.112a | 6.429±0.566a |  |
|  | WT+Ta536+Aa | 1.799±0.236d | 0.344±0.100f | 0.687±0.112d |  |
|  | OX1+Ta536+Aa | 2.599±0.145c | 0.840±0.027e | 2.159±0.177b |  |
| *PdPapIAA6-1* | WT | 1.000±0.000cd | 1.000±0.000cd | 1.000±0.000d |  |
|  | OX1 | 1.440±0.246b | 1.042±0.115cd | 2.144±0.132c |  |
|  | WT+Ta536 | 0.764±0.073de | 1.455±0.250b | 1.409±0.164cd |  |
|  | OX1+Ta536 | 2.731±0.169a | 1.154±0.211bc | 1.691±0.152cd |  |
|  | WT+Aa | 0.519±0.096e | 0.730±0.158d | 2.096±0.188cd |  |
|  | OX1+Aa | 2.663±0.168a | 2.549±0.357a | 11.234±1.361a |  |
|  | WT+Ta536+Aa | 0.877±0.125d | 0.373±0.092e | 1.522±0.428cd |  |
|  | OX1+Ta536+Aa | 1.170±0.105c | 0.717±0.102d | 4.022±0.787b |  |
| *PdPapIAA6-2* | WT | 1.000±0.000d | 1.000±0.000d | 1.000±0.000d |  |
|  | OX1 | 0.366±0.006d | 7.113±1.632b | 15.107±0.432c |  |
|  | WT+Ta536 | 0.968±0.340d | 2.622±0.343cd | 3.352±0.339d |  |
|  | OX1+Ta536 | 9.142±1.445b | 13.308±0.938a | 13.120±0.742c |  |
|  | WT+Aa | 0.196±0.024d | 0.229±0.055d | 1.097±0.079d |  |
|  | OX1+Aa | 11.953±0.845a | 13.596±1.133a | 50.222±3.958a |  |
|  | WT+Ta536+Aa | 1.250±0.142d | 0.327±0.048d | 0.353±0.029d |  |
|  | OX1+Ta536+Aa | 6.513±0.561c | 4.832±0.937bc | 36.215±6.279b |  |

^1^ Different lowercase letters represent significant differences between the samples of each poplar compartment undergone different treatments. All significances were at *P* < 0.05.
