# Supplementary material for: AUXIN RESPONSE FACTOR 1 Acts as a Positive Regulator in the Response of Poplar to Trichoderma asperellum Inoculation in Overexpressing Plants
Source: Plants (Basel). 2020 Feb 19;9(2):272. doi: 10.3390/plants9020272 (PMC7076496; doi:10.3390/plants9020272)
Supplement: Supplementary file 1 [file plants-09-00272-s001.zip › supplementary material/Supplementary Figure 1 revised Round 2.docx]

| 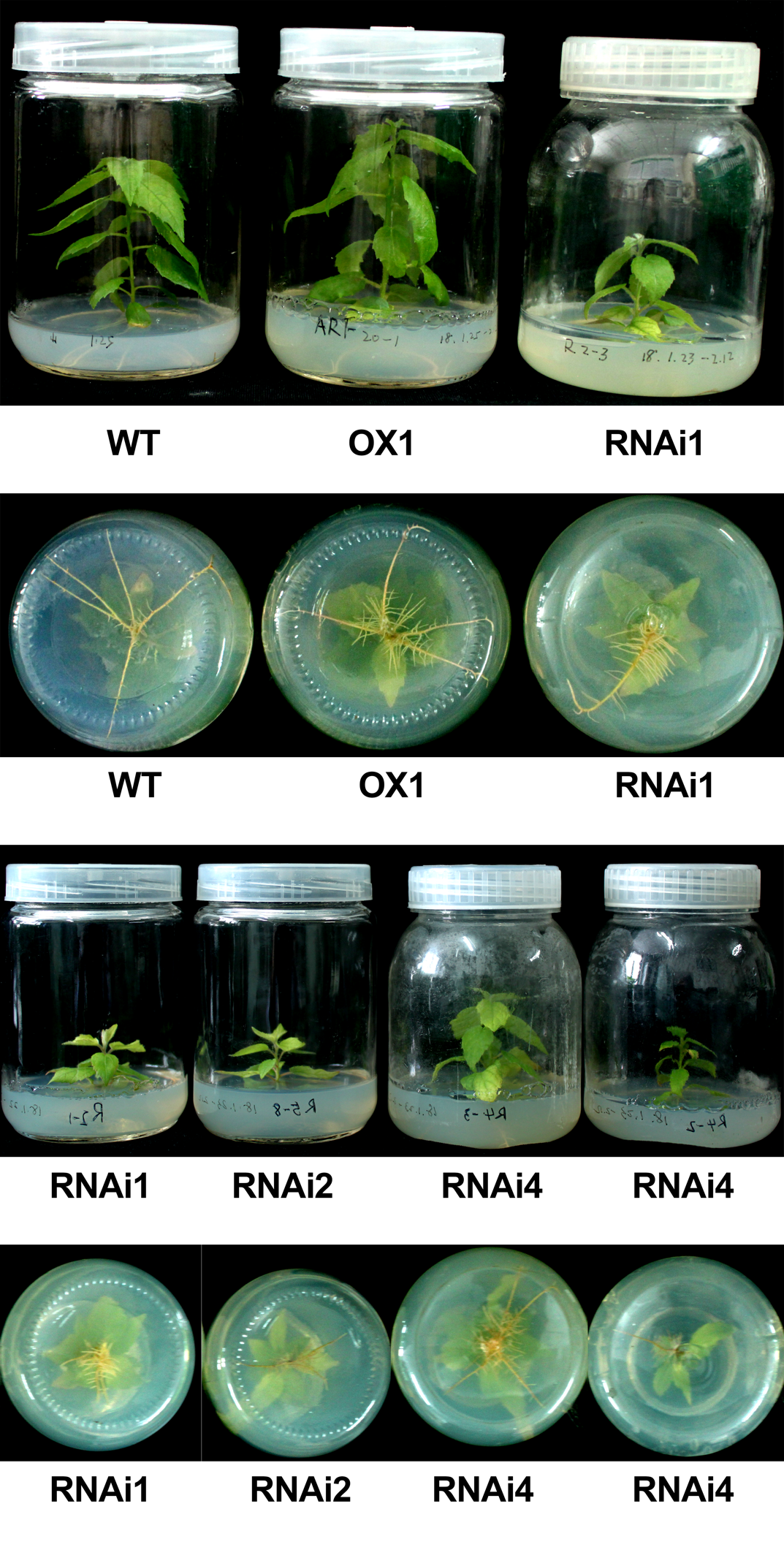 |
| --- |

**Supplementary Figure S1.** The phenotypes of *PdPapARF1* RNAi poplar plants compared to a wild-type plant and an OX1 plant. Three-week-old subcultured poplar plants in 8 cm (bottom diameter) vessels and their roots seen from the bottom of the vessels are shown. Repeated labels represent different plant clones of the same transformed poplar line. The difference of the phenotypes of plant clones of the same line indicates the possible individual difference. These data are only provided as supplementary material because our focus is on the positive effects of *PdPapARF1* and did not conduct further studies on the RNAi plants.
