# Supplementary material for: AUXIN RESPONSE FACTOR 1 Acts as a Positive Regulator in the Response of Poplar to Trichoderma asperellum Inoculation in Overexpressing Plants
Source: Plants (Basel). 2020 Feb 19;9(2):272. doi: 10.3390/plants9020272 (PMC7076496; doi:10.3390/plants9020272)
Supplement: Supplementary file 1 [file plants-09-00272-s001.zip › supplementary material/Supplementary material 1 revised Round 2.docx]

>KM113035.1

ATGAATCATACCTCCGGAGGAAACCCACATCCAGGGGGATGCAATGATGCTTTGTACAAAGAACTATGGCATGCCTGTGCTGGACCTCTTGTCACTCTTCCTTGTGAAGGGGAGCGAGTGTATTATTTTCCTCAAGGTCACATGGAACAGCTTGAAGCATCTATGCATCAGGGGATGGAGCAGCAAATGCCCTCATTTAACCTTCCATCTAAAATTCTGTGTAAAGTTGTTAATGTACAGCGCAGGGCTGAACCTGAAACAGATGAAGTTTATGCCCAGATAACACTGCTTCCTGAACCAGATCAAAGCGAGGTTACGAGTCCTGATCCTCCACTCCCAGAACCTGAAAGATGCGCGGTCCATTCATTTTGCAAGACACTTACTGCTTCTGACACAAGCACTCATGGTGGTTTCTCTGTTCTTCGAAGGCATGCAGATGATTGTCTGCCACCTTTGGATATGTCTCAGCAGCCACCTTGGCAGGAATTGGTTACAACTGATCTGCATGGCAATGAGTGGCATTTTCGACACATTTTTCGAGGTCAACCAAGGCGTCACCTGCTCACAACAGGGTGGAGTGTCTTTGTTAGCTCAAAAAAGTTAGTTGCGGGTGATGCATTCATCTTCCTAAGGGGAGAAAATGGCGAGCTTCGTGTGGGAGTAAGGAGGCTCATGAGGCAACAGACAAATATGCCATCTTCTGTTATATCTAGCCAAAGCATGCATCTAGGGGTTCTTGCTACTGCTTCTCATGCCATTGCGACTGGAACCCTTTTTTCTGTCTTCTACAAGCCAAGAACAAGTAGGTCTGAGTTCATTGTAAATCTTAACAAGTATATTGAAGCTCAAAACCACAAGCTTTCTGTAGGGATGAGGTTTAAGATGAGATTTGAGGGTGAGGAAGTTCCTGAACGAAGGTTTAGTGGCACCATTGTTGGCGTTGGAGATGATATATCATCGGGATGGGCTGATTCTGAGTGGAGATCATTAAAGATCCATTGGGATGAACCTTCTTCCATCTTGCGTCCAGAGAGAGTATCACCATGGGATTTGGAACCTCTTGTTGCAACTACTCCTTCGAACTCCCAACCTATGCAGAGGAACAAGCGGCCACGGCCATCTGTCTTGCCCTCACCAACAGCCGATCTTTCTGCACTTGGTATTTGGAAACCTTCGGTTGAGTCTTCGGCTTTCGCATATGGTGAATCACAACGTGGACGAGACCCTTATCCATCACCCAATTTCTCTACCACTGCAAAGGCCAACTCTCTTAGCTTCTGTGGCAATAGTCAAGTGACCAGTGTTTCGCCGAATTCAATGTATCGGCCTAACCAAGTGGAAAGTGTCACAGATTCGTTTGCTCCAGTTGTAAACAAAGATTTGGGAGAAAGGAGACAGGGCACTGGGATTGGCTACAGACTTTTCGGGATTCAACTTATTGACAATTTCAATTCAGAAGGAACTTCACCAGTTGTTACTTTGTCTGGAACAGTGGGCAATGATCGCCCAGTTGTGTCTTTGGAGGCCGAGTCTGATCAGCATTCTGAGCCTGAGAAATCATGTCTGAGATCTCCTCAGGAGTTGCAAAGTAGGCAAATCAGGAGCTGCACAAAGGTTCACATGCAAGGCGTTGCTGTTGGAAGAGCTGTTGATTTGACACAGTTTGAGCGCTATGAAGACCTGCTGAGGAAGCTGGAGGAGATGTTTGATATCGAAGGTGAGCTCAGTGGATCCACAAAGAAATGGCAGGTTGTGTATACTGATAATGAAGATGACATGATGAAGGTTGGAGATGATCCATGGCATGAGTTCTGCAGCATGGTGAAGAAGATTTTTATCTATGCATCTGAAGAAGTCAAGAGGCTGTCACCCAAGATTAAACTTTCAGGTGATGAAGAGATCAAAGGAGGTAGTGCCAGTGCTAATGCTGATGCAGCTGTTAACACAGAGGACCGCTCATCTATCGTTGGGCCTGGATGCTGA

*Note*: this is the cds of *PdPapARF1*. The black box marks the specific fragment we used for constructing the RNAi vector.
