# Supplementary material for: AUXIN RESPONSE FACTOR 1 Acts as a Positive Regulator in the Response of Poplar to Trichoderma asperellum Inoculation in Overexpressing Plants
Source: Plants (Basel). 2020 Feb 19;9(2):272. doi: 10.3390/plants9020272 (PMC7076496; doi:10.3390/plants9020272)
Supplement: Supplementary file 1 [file plants-09-00272-s001.zip › supplementary material/Table S2 revised.docx]

**Table S2.** Data for Figure 4.

| **Hormone** | **Treatment** | **ST** | **L** | **R** |  |
| --- | --- | --- | --- | --- | --- |
| **IAA concentration (ng/g FW^1^)** | WT | 16.463±2.499d^2^ | 10.757±0.042cd | 7.223±0.231d |  |
|  | OX1 | 42.327±0.100b | 31.503±1.766d | 22.354±2.081c |  |
|  | WT+Ta536 | 16.732±0.746d | 9.337±0.846d | 9.881±1.305b |  |
|  | OX1+Ta536 | 50.198±2.430a | 11.695±1.866d | 8.223±1.027a |  |
|  | WT+Aa | 19.239±4.715d | 7.970±0.338b | 8.828±0.072c |  |
|  | OX1+Aa | 36.075±1.329c | 12.144±3.479cd | 7.741±0.682b |  |
|  | WT+Ta536+Aa | 18.134±3.314d | 12.802±2.899cd | 10.937±2.540c |  |
|  | OX1+Ta536+Aa | 47.802±4.445ab | 25.977±4.346a | 16.057±2.526b |  |
| **JA concentration (ng/g FW)** | WT | 108.002±2.137c | 63.623±12.760cd | 223.017±11.495d |  |
|  | OX1 | 267.925±13.051b | 46.597±0.808d | 374.794±16.862c |  |
|  | WT+Ta536 | 79.090±6.268c | 46.123±9.543d | 493.245±70.169b |  |
|  | OX1+Ta536 | 381.878±11.350a | 37.107±4.931d | 686.248±36.267a |  |
|  | WT+Aa | 116.712±16.688c | 112.875±2.955b | 364.848±12.149c |  |
|  | OX1+Aa | 389.033±24.167a | 81.954±15.962c | 516.719±20.883b |  |
|  | WT+Ta536+Aa | 116.239±13.334c | 81.102±16.755c | 340.084±30.141c |  |
|  | OX1+Ta536+Aa | 291.852±30.628b | 367.323±27.461a | 534.935±34.737b |  |
| **SA concentration (ng/g FW)** | WT | 4896.004±322.736b | 3653.474±225.172a | 2948.453±59.263d |  |
|  | OX1 | 5383.659±618.789ab | 2118.893±121.509c | 5702.048±216.941b |  |
|  | WT+Ta536 | 5511.427±209.662ab | 4326.453±129.993a | 4487.008±501.419c |  |
|  | OX1+Ta536 | 5753.621±78.504ab | 2889.828±226.053b | 6825.072±1004.462a |  |
|  | WT+Aa | 3992.982±304.288c | 4083.341±101.258a | 3402.854±343.429d |  |
|  | OX1+Aa | 5795.411±474.785ab | 4333.182±470.196a | 6213.880±182.353ab |  |
|  | WT+Ta536+Aa | 5825.950±81.798a | 4135.647±443.055a | 3103.603±65.686d |  |
|  | OX1+Ta536+Aa | 6020.890±599.256a | 4341.495±682.468a | 5984.456±196.175ab |  |

^1^ Fresh weight. ^2^ Different lowercase letters represent significant differences between the samples of each poplar compartment undergone different treatments. All significances were at *P* < 0.05.
