# Supplementary material for: AUXIN RESPONSE FACTOR 1 Acts as a Positive Regulator in the Response of Poplar to Trichoderma asperellum Inoculation in Overexpressing Plants
Source: Plants (Basel). 2020 Feb 19;9(2):272. doi: 10.3390/plants9020272 (PMC7076496; doi:10.3390/plants9020272)
Supplement: Supplementary file 1 [file plants-09-00272-s001.zip › supplementary material/Table S1 revised Round 2.docx]

**Table S1.** Data for Figure 2d.

| **Plant height**  **(cm)** | **Treatment** | **10 d**^1^ | **20 d** | **30 d** | **40 d** |
| --- | --- | --- | --- | --- | --- |
|  | WT | 8.100±0.100de^2^ | 13.267±0.058g | 19.233±0.208g | 29.033±0.153f |
|  | OX1 | 9.433±0.115c | 16.333±0.058a | 25.867±0.115a | 38.400±0.100a |
|  | WT+Ta536 | 8.267±0.058d | 13.100±0.100g | 21.200±0.200f | 31.733±0.115d |
|  | OX1+Ta536 | 9.867±0.115b | 15.667±0.153c | 24.267±0.252b | 38.900±0.100a |
|  | WT+Aa | 8.233±0.058de | 13.633±0.115f | 21.867±0.153e | 29.333±0.577f |
|  | OX1+Aa | 10.133±0.115a | 15.867±0.153b | 21.800±0.200e | 33.600±0.361c |
|  | WT+Ta536+Aa | 8.033±0.153e | 14.267±0.153e | 22.433±0.058d | 30.533±0.416e |
|  | OX1+Ta536+Aa | 9.367±0.153c | 15.467±0.058d | 23.533±0.058c | 36.567±0.153b |
| **Leaf**  **count** | WT | 15.333±0.577d | 21.000±1.000b | 22.667±1.155d | 26.667±0.577c |
|  | OX1 | 18.000±1.000c | 25.333±0.577a | 31.000±1.000a | 30.667±0.577b |
|  | WT+Ta536 | 18.667±0.577c | 21.333±0.577b | 24.333±0.577cd | 27.333±0.577c |
|  | OX1+Ta536 | 20.333±0.577b | 26.333±0.577a | 30.000±1.000a | 32.667±0.577a |
|  | WT+Aa | 18.667±0.577c | 21.667±1.155b | 24.333±0.577cd | 25.100±0.100d |
|  | OX1+Aa | 21.333±0.577ab | 26.667±0.577a | 27.667±0.577bc | 31.333±0.577b |
|  | WT+Ta536+Aa | 20.667±0.577b | 25.333±0.577a | 25.667±0.577cd | 26.667±0.577c |
|  | OX1+Ta536+Aa | 22.333±0.577a | 26.333±0.577a | 25.000±5.196cd | 32.333±0.577a |

^1^ d, day(s). ^2^ Different lowercase letters represent significant differences between the samples of different treatments on the same day. All significances were at *P* < 0.05.
