# Supplementary material for: AUXIN RESPONSE FACTOR 1 Acts as a Positive Regulator in the Response of Poplar to Trichoderma asperellum Inoculation in Overexpressing Plants
Source: Plants (Basel). 2020 Feb 19;9(2):272. doi: 10.3390/plants9020272 (PMC7076496; doi:10.3390/plants9020272)
Supplement: Supplementary file 1 [file plants-09-00272-s001.zip › supplementary material/Table S4 revised Round 2.docx]

**Table S4.** All the primers used in this study.

| **Purpose or resulted data** | **Primer name** | **Primer sequence** |
| --- | --- | --- |
| Cloning *PdPapARF1* | *PdPapARF1* | F: ATGAATCATACCTCCGGAGG |
|  |  | R: TCAGCA TCCAGGCCCAACGA |
| Constructing the OX vector | *PdPapARF1-*OX | F: GGACTCTAGAGGATCCCCATGAATCATACCTCCGGAGGAAAC |
|  |  | R: AATTCGAGCTCGGTACCCTCAGCATCCAGGCCCAACGATAG |
| Constructing the RNAi vector | *PdPapARF1-*RNAi-F | F: CCCAATCGATGATTTGTCTTCTACAAGCCAAGAAC |
|  |  | R: AATTCTTACACATTTAATGATCTCCACTCAGAATC |
|  | *PdPapARF1-*RNAi-R | F: AGTTAATTAAGACCCACTCAGACCTACTTGTTCTTG |
|  |  | R: CTAGGGACTAGTCCCCTCACAACAGGGTGGAGTGT |
| Examining *PdPapARF1* cds insertion | *PdPapARF1-*ins | F: GATGACGCACAATCCCACTATC |
|  |  | R: ATGTGCTGCAAGGCGATTAAG |
| Examining *PdPapARF1* RNAi fragment insertion | *PdPapARF1-*RNAi*-*ins | F: GAAGTCCAGCTGCCAGAAAC |
|  |  | R: CACCAAATCGAATCGATGAG |
| Determining relative gene expression | *PdPapARF1* | F: TGCCATTGCGACTGGAACCC |
|  |  | R: CCACTCAGAATCAGCCCATCCC |
|  | *PdPapCOI1* | F: GGCTCAAGGCTGCTTAGAAC |
|  |  | R: CTCGAACTCCATTGTCAAGG |
|  | *PdPapJAZ5* | F: GTCATTGGCACCACTGATC |
|  |  | R: TCTGAGTCCCAGAAGAGCC |
|  | *PdPapMYC2* | F: GTGGGTTACCTGGTCAAGC |
|  |  | R: CCTTATTCATCAGATCCGAGC |
|  | *PdPapNPR1* | F: CTCTAGAACAGTGGAACTTGG |
|  |  | R: ATCTTCATTGAATGCCTTGC |
|  | *PdPapTGA* | F: AGCCTCTTATCAACCGTGTTC |
|  |  | R: CCTCAAGCTGATGACCTGAC |
|  | *PdPapPR1* | F: GGTGAGAACATTGCTGAAGG |
|  |  | R: CTTGCACTTAGCTCTGCCAC |
|  | *PdPapARF6* | F: TCCTTCGGAATTTGTCATTC |
|  |  | R: CCGAGCAGGATCTAAATCAC |
|  | *PdPapARF8* | F: AGTGATCTGGATCCTGTTCG |
|  |  | R: TGGATACATGGGGAAAGTTG |
|  | *PdPapGH3.5* | F: GCAGGCTATGTCAGTTCACG |
|  |  | R: TGCACCTTGTTGTTCATGG |
|  | *PdPapGH3.6* | F: AATTCAATCACTGCACCGAC |
|  |  | R: TGAATTGAGGAGCCTTGTTC |
|  | *PdPapPIN1-1* | F: GTTTAATGGTGCAAGTTGTGG |
|  |  | R: GAGGCTGTCTACCATCAAGTG |
|  | *PdPapPIN1-2* | F: TATGGGCATTCCTTTGCTC  R: AGTGTCTGGGAATTGCTCAG |
|  | *PdPapLAX3* | F: TGTTCAAGAGAGCTCTGGC |
|  |  | R: GAGAAAGTGACCATGTGTGC |
|  | *PdPapIAA6-1* | F: GGCAAAGCCTTGAAGGATAC |
|  |  | R: CCTTGGCTTCTGACCTCTTC |
|  | *PdPapIAA6-2* | F: GAATTGGCAAAGCCTTGAAG |
|  |  | R: TGAAATTCCCTTAAGAGCGC |
|  | *PdPapACT1* | F: GCTGAGAGATTCCGTTGCCCTG  R: GGCGGTGATCTCCTTGCTCATT |
|  | *PdPapEF1-α* | F: TGGGTCGTGTTGAAACTGGTGT  R: GGCAGGATCGTCCTTGGAGTTC |
|  | *PdPapUBQ* | F: TGTTGTGATCAACGCGAACTCG  R: GAGGATGCCTAGTGCTACGCAT |
